# Differential Effects of Halofuginone Enantiomers on Muscle Fibrosis and Histopathology in Duchenne Muscular Dystrophy

**DOI:** 10.3390/ijms22137063

**Published:** 2021-06-30

**Authors:** Sharon Mordechay, Shaun Smullen, Paul Evans, Olga Genin, Mark Pines, Orna Halevy

**Affiliations:** 1 Department of Animal Sciences, The Hebrew University of Jerusalem, P.O. Box 12, Rehovot 76100, Israel; sharon.mordechay@gmail.com; 2 Center for Synthesis and Chemical Biology, School of Chemistry, University College Dublin, D04 N2E5 Dublin, Ireland; shaun.smullen@liverpoolchirochem.com (S.S.); paul.evans@ucd.ie (P.E.); 3 The Volcani Center, Institute of Animal Science, Bet Dagan 52505, Israel; olga.genin1@mail.huji.ac.il (O.G.); mark.pines@mail.huji.ac.il (M.P.)

**Keywords:** skeletal muscle, Duchenne muscular dystrophy, fibrosis, utrophin, halofuginone, stereoisomer, febrifugine

## Abstract

Progressive loss of muscle and muscle function is associated with significant fibrosis in Duchenne muscular dystrophy (DMD) patients. Halofuginone, an analog of febrifugine, prevents fibrosis in various animal models, including those of muscular dystrophies. Effects of (+)/(−)-halofuginone enantiomers on motor coordination and diaphragm histopathology in *mdx* mice, the mouse model for DMD, were examined. Four-week-old male mice were treated with racemic halofuginone, or its separate enantiomers, for 10 weeks. Controls were treated with saline. Racemic halofuginone-treated mice demonstrated better motor coordination and balance than controls. However, (+)-halofuginone surpassed the racemic form’s effect. No effect was observed for (−)-halofuginone, which behaved like the control. A significant reduction in collagen content and degenerative areas, and an increase in utrophin levels were observed in diaphragms of mice treated with racemic halofuginone. Again, (+)-halofuginone was more effective than the racemic form, whereas (−)-halofuginone had no effect. Both racemic and (+)-halofuginone increased diaphragm myofiber diameters, with no effect for (−)-halofuginone. No effects were observed for any of the compounds tested in an in-vitro cell viability assay. These results, demonstrating a differential effect of the halofuginone enantiomers and superiority of (+)-halofuginone, are of great importance for future use of (+)-halofuginone as a DMD antifibrotic therapy.

## 1. Introduction

Muscular dystrophies (MDs) are a heterogeneous group of genetic disorders characterized by progressive loss of muscle strength and integrity. The most common of these is Duchenne MD (DMD), which is caused by genetic aberrations of dystrophin, leading to a complete absence or a strong decrease in dystrophin levels. Disease onset is in early childhood, and it is characterized by the presence of high levels of muscle creatine kinase, muscle hypertrophy and necrotic myofibers. This is accompanied by repeated muscle degeneration–regeneration cycles and progressive replacement of the contractile muscle tissue with collagenous (mainly collagen type I) fibrotic tissue [1,2]. Progressive muscle weakness leads to loss of ambulation by 10–12 years of age, and death from respiratory or cardiac failure in the twenties [3]. Fibrosis is evident mainly in the diaphragm, the most affected muscle at early stages of DMD, and its prevention enhances muscle repair and function [4]. A 395-kDa protein, utrophin, shares a high degree of homology with dystrophin and its level increases during early stages of DMD [5,6,7]. Recent studies have reported that utrophin is expressed in an inverse relationship with fibrosis in muscles of DMD patients [8] and in the diaphragm of *mdx* mice, a mouse model of DMD [8,9].

Currently, DMD patients are mainly treated with glucocorticoids [10,11]. However, several new therapeutics have recently been developed to target the disease. For example, gene therapy such as, viral-delivery of micro-dystrophin [12], antisense oligonucleotide-mediated exon skipping [13], or injection of specific antibodies to myostatin, a member of the TGFβ family which acts in a similar manner to TGFβ [14,15]. 

**Figure 1:**
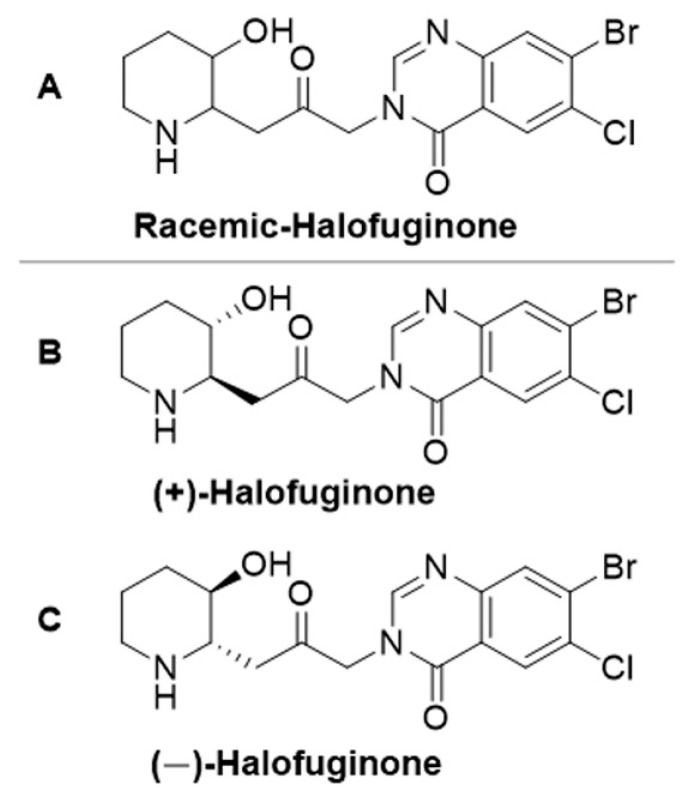
Schematic representation of racemic halofuginone (**A**) and its optical enantiomers (+)-halofuginone (**B**), and (−)-halofuginone (**C**).

Halofuginone{7-bromo-6-chloro-3-[3-(3-hydroxy-2-piperidinyl)-2-oxopropyl]-4(3*H*)-quinazolinone} is a halogenated analog of the naturally occurring quinazoline-type alkaloid (+)-febrifugine (Figure 1) isolated from the plant *Dichroa febrifuga* [16,17]. Halofuginone has been shown to act as an antifibrotic agent in numerous diseases in which an increase in collagen is the major cause of the disease [4,18]. Halofuginone inhibits fibroblast conversion to myofibroblasts by inhibiting Smad3 phosphorylation downstream of transforming growth factor β (TGFβ), thereby preventing Smad3 entrance into the nucleus and transcription of the collagen type I gene [19,20,21]. Halofuginone has been reported to inhibit fibrosis in mouse models of DMD, congenital MD (CMD) and dysferlinopathy, regardless of the cause of the disease [22,23,24,25]. It reduces tissue inflammation and infiltration of myofibroblasts—which are typical symptoms of MDs. In addition, halofuginone increases mean myofiber diameter and muscle hypertrophy [22,23,24,25]. Some of these effects have been recently explained by the elevation of utrophin and reduction of moesin in mouse models for DMD and CMD in response to halofuginone treatment [8,26]. The mechanism of action of halofuginone can be explained, at least in part, by its inhibitory effect on prolyl-tRNA synthetase activity, as was recently shown in Th_17_ cells [27,28]. The hydroxy group on halofuginone’s piperidinyl ring plays a key role in this inhibition [29]. Indeed, a recent study of ours demonstrated that deoxyhalofuginone, a halofuginone analog in which the hydroxy group has been removed from the piperidinyl ring [30], blocked the positive effects of halofuginone on fibrosis and muscle histopathology in *mdx* mice [9]. Recently, halofuginone have been shown to directly affect myoblast fusion in the mouse models for DMD and dysferlinopathy [19,25] and improve membrane repair in dysferlinopathy, probably via synaptotagmin-7 compensation [31]. 

Halofuginone was not specifically developed to treat fibrosis or muscle dysfunction in MDs. Moreover, in animal models and humans, when provided in excess doses, halofuginone possesses adverse and even toxic side effects [32,33,34]. In addition, halofuginone has always been utilized in its racemic form and its single enantiomers have never been tested in MDs. An earlier study showed that enantiomeric forms of halofuginone lactate exhibit different activity and toxicity against *Cryptosporidium parvum*—a parasite which causes diarrhea in newborn calves [35]—in mice [36]. We previously reported the de-novo synthesis of the two optically active enantiomers of halofuginone (Figure 1) [37], and our hypothesis is that the single enantiomers of halofuginone affect muscle function and histopathology of *mdx* mice differently. In the present study, these separate enantiomers, as well as the racemic form of halofuginone, were evaluated in relation to their effects on motor coordination, fibrosis and overall histopathology in diaphragms of *mdx* mice.

## 2. Results

### 2.1. Motor Coordination

**Figure 2:**
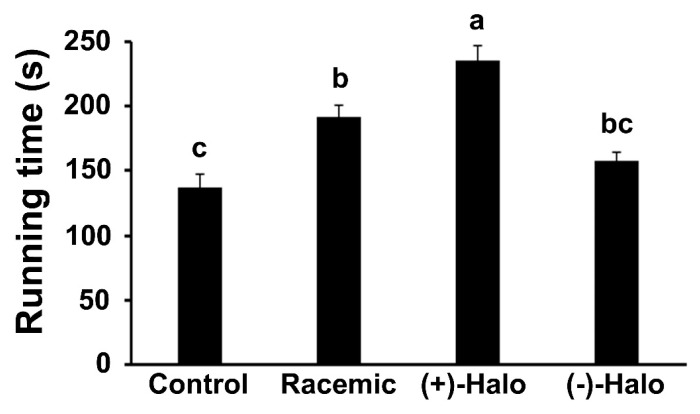
Effect of the racemic halofuginone (Racemic), (+)-halofuginone enantiomer [(+)-Halo] and (−)-halofuginone enantiomer [(−)-Halo] on the motor coordination of *mdx* mice at 14 weeks of age. Data are presented as mean + SEM (*n* = 4–6/group). Bars with different letters indicate significant difference between groups (*p* < 0.05).

From the age of 4 weeks, mice were injected for a period of 10 weeks with saline (Control), the separate (+) or (−) halofuginone enantiomers [(+)-Halo and (−)-Halo, respectively], or a combination of equal amounts of each enantiomer (Racemic). The racemic combination represents the halofuginone form used in our previous experiments and in clinical trials [34,38]. At the end of the experiment, the 14-week-old mice were subjected to a motor coordination test as represented by the RotaRod running time (*n* = 4–6 mice per group). Administration of the racemic molecule significantly enhanced the running time of the Racemic group on the RotaRod compared to that of the Control group (Figure 2). However, the (+)-Halo group surpassed this effect, with a running time that was more than 1.7-fold that of the Control group. The running time of the (−)-Halo mice remained as low as that of the Control group.

### 2.2. Sirius Red Staining

**Figure 3:**
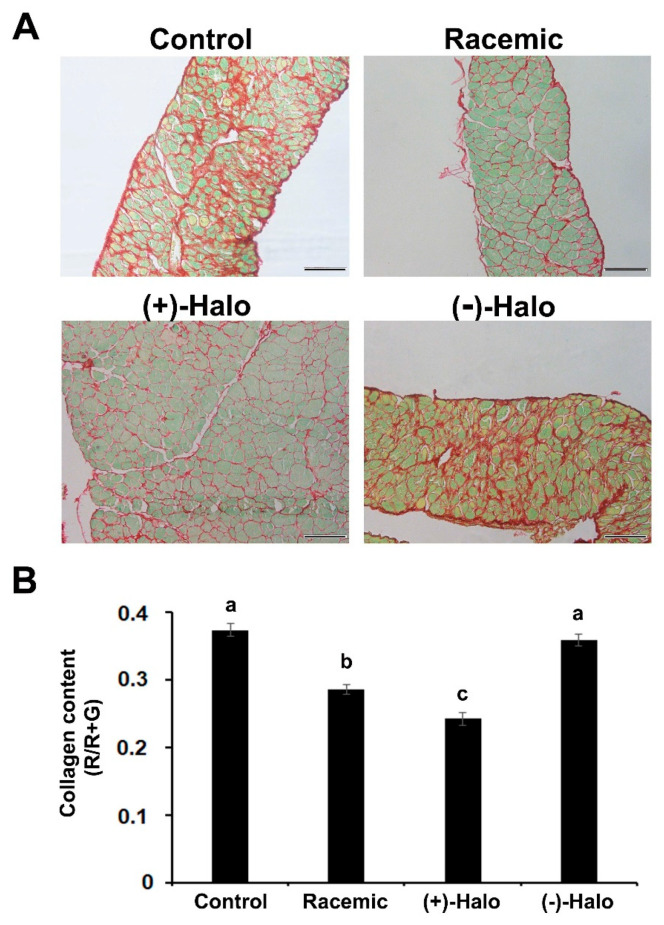
Collagen content in diaphragms of *mdx* mice at 14 weeks of age. (**A**) Sirius red staining of diaphragm sections derived from mice treated for 10 weeks with saline (Control), (+)-halofuginone enantiomer [(+)-Halo], (-)-halofuginone enantiomer [(-)-Halo] or their racemic combination (Racemic; 10 μg/3 times per week). Note the high levels of red staining representing the fibrillar collagens (types I and III) in the Control and (-)-Halo groups against the large reduction in the Racemic and (+)-Halo groups. Bar, 100 μm. (**B**) Quantitative analysis of collagen levels in the diaphragms. The proportion of the collagen content was calculated as red area (R) divided by the total area (red + green, R + G) in 20 pictures from each of 3 different mice (*n* = 60) and presented as (mean arbitrary units ± SEM). Bars with different letters indicate significant difference between groups (*p* < 0.05).

The fibrotic tissue, which mainly consists of collagen type I, was very prominent in the diaphragms of both the Control and (−)-Halo groups (Figure 3A, red stain). The myofibers were surrounded by large bundles of collagen, clearly demonstrating their advanced dystrophic state. As expected, injection of the racemic molecule for 10 weeks reduced the collagen content—as can be observed by only a thin red stain remaining between and around the myofibers. Quantification analysis revealed that in the Racemic group, the collagen content was significantly lower than in the Control group (Figure 3B). Injection of the (+)-halofuginone enantiomer further reduced these levels, and they were significantly lower than those found in the Racemic group. No difference in collagen levels was found in the (−)-halofuginone-injected mice compared to controls.

### 2.3. Morphometric Analyses

**Figure 4:**
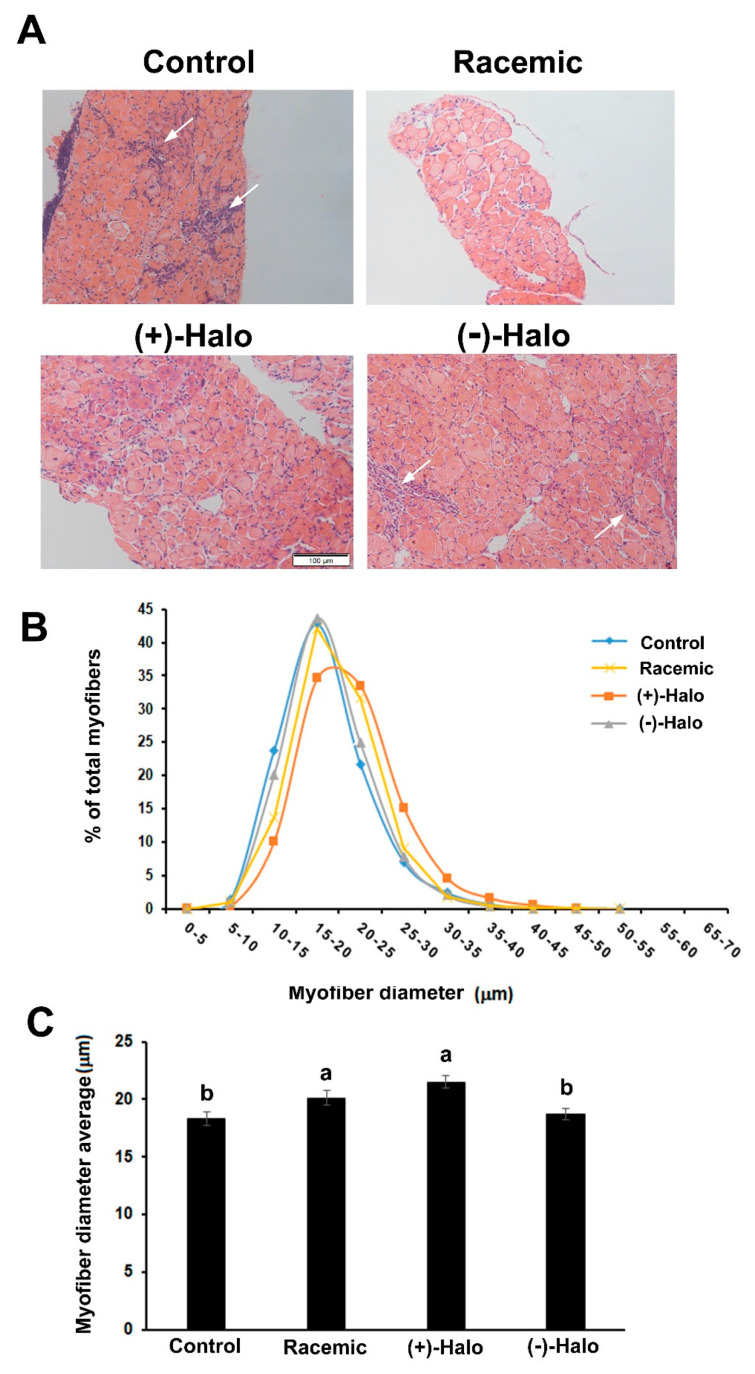
Effect of the halofuginone enantiomers and their racemic combination on the histopathology of *mdx* diaphragms. The experimental course was as described in Figure 3. (**A**) Hematoxylin and eosin (H&E) staining of diaphragms derived from the treated mice at 14 weeks of age. Fewer degeneration areas (white arrows) are observed in the Racemic and (+)-halofuginone enantiomer [(+)-Halo] sections with a more unformed appearance of the myofibers. Bar, 100 μm. (**B**) Distribution of myofiber diameters in *mdx* diaphragms. Myofibers were clustered in bin intervals of 0.5 μm, and data are presented as percentage of total myofibers. Between 4000 to 4700 myofibers were counted for each treatment group. (**C**) Quantitation analysis the average of myofiber diameter of the experimental groups, presented as mean ± SE (*n* = 4). Bars with different letters indicate significant difference between treatments (*p* < 0.05).

Hematoxylin and eosin (H&E) staining of the diaphragm sections sampled from the experimental groups showed their degenerative state in the Control group. Specifically, areas of infiltrated cells were observed that represented areas of inflammation, as well as numerous irregular myofibers (Figure 4A). A very similar picture was observed in the (−)-halofuginone-injected mice. In contrast, in diaphragm sections derived from the racemic and (+)-halofuginone-injected mice, there were less inflamed regions and less irregular myofibers. Measurements of myofiber diameter—a parameter of myofiber hypertrophy—in the H&E-stained diaphragm sections revealed distribution of the myofibers spanning between 0 and 55 μm in all diaphragms (Figure 4B). However, whereas the distribution of the myofiber diameters was identical in the Control and (−)-Halo groups, with a peak at around 16 μm, the myofiber diameters in the Racemic group, and even more so in the (+)-Halo group, shifted to the right to the higher bins. The average myofiber diameters in both Control and (−)-Halo groups were similar and lower compared to the Racemic and (+)-Halo groups (Figure 4C).

### 2.4. Cytotoxicity Assay in C2 Myogenic Cells

Cytotoxicity of the separate enantiomers and the racemic halofuginone was determined in myogenic C2 cells [39] by evaluating the activity levels of lactate dehydrogenase (LDH) in the medium. Cells were treated with the racemic halofuginone or its separate enantiomers in growing medium for 24 h. A concentration range of 0–10 ng/mL was chosen in accordance with our previous publications in which 10 ng/mL of racemic halofuginone had a promotive effect on muscle cells [19,40,41]. Following the incubation, the cells were evaluated for LDH levels with a commercial kit (CyQUANT). The LDH levels were calculated as percentage of the maximal LDH activity (i.e., maximal toxicity, 100%) found in cells that were treated with lysis buffer as described in Section 4.7. Control, untreated cells presented very low toxicity of 3.4 ± 0.24% (average ± SEM of three independent experiments). In the treated cells, the percentage of toxicity remained low and was either comparable to the control levels, or slightly higher, in the range of 3.44 ± 0.46 to 5.65 ± 1.05%.

### 2.5. Utrophin Expression 

**Figure 5:**
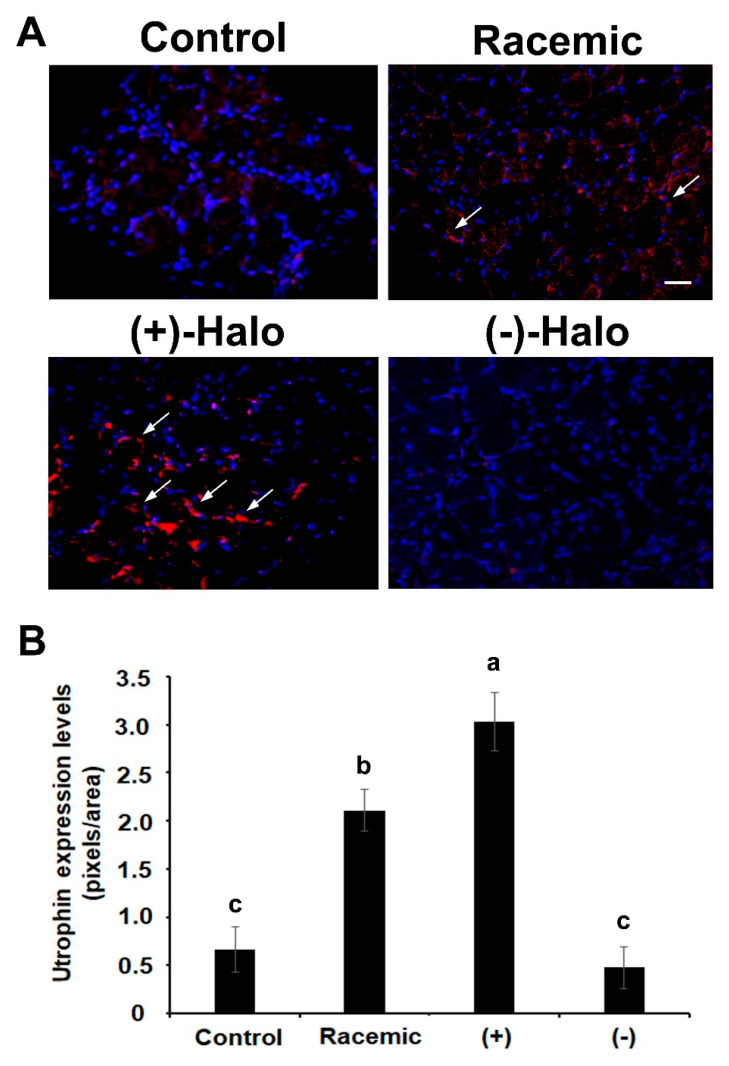
Utrophin levels in the experimental *mdx* mice at the age of 14 weeks. (**A**) Immunostaining of diaphragm sections derived from *mdx* mice with an antibody against utrophin (red staining, white arrows). Nuclei were stained with DAPI (blue). The sections were visualized by confocal microscopy. Bar, 50 μm. (**B**) Quantification analysis of utrophin levels was performed on images of three mice with 10 sections/diaphragm (*n* = 30). The results are presented as mean pixels/unit area ± SE. Bars with different letters indicate significant difference between treatments (*p* < 0.05).

Utrophin levels, that were demonstrated to increase in *mdx* mice when compared to wild-type were further raised when mice were treated halofuginone [8]. Here, very low positive staining for utrophin was observed in the diaphragms of the Control mice at 14 weeks of age (Figure 5A). An increase in utrophin-positive myofibers was observed in the diaphragms of the racemic- as well as in the (+)-halofuginone-treated mice (Figure 5A, white arrows). However, hardly any myofibers with utrophin staining were observed in the diaphragms of mice that were treated with the (−)-halofuginone enantiomer. A quantitation analysis revealed that the increase of utrophin staining was fourfold higher in Racemic than in the Control group (Figure 5B). The staining in the (+)-Halo group significantly exceeded that of the Racemic group and was almost six-fold higher than in the Control group. The utrophin staining remained as low as in the Control group (Figure 5B).

## 3. Discussion

Halofuginone has proven to be very effective as an antifibrotic drug, and to alleviate impaired skeletal muscle function and improve pathology markers in various animal models for MDs [9,22,23,24,25]. Those studies, however, all used racemic halofuginone. Our technique of synthesizing separate optically active enantiomers of halofuginone [37] allowed us, for the first time, to examine the effects of optically active halofuginone on motor coordination and running time and histopathology. This was determined by administering each halofuginone enantiomer separately to *mdx* mice—the benchmark mouse model for DMD. The results demonstrated a differential effect of the two halofuginone enantiomers on the diaphragm fibrosis and histopathology of *mdx* mice. A clear effect was also observed on relative motor coordination. The (+)-enantiomer was active, whereas the (−)-enantiomer presented results equivalent to the saline-treated control. Moreover, the (+)-enantiomer was superior to the racemic halofuginone in improving fibrosis and motor coordination.

In *mdx* mice treated for 10 weeks, from the age of 4 weeks, administration of the two optical enantiomers significantly differed in all analyzed parameters: motor coordination and balance, fibrosis and degeneration of the diaphragm, myofiber diameter, and utrophin expression levels. In all cases, the (+)-enantiomer of halofuginone had a more positive effect than its (−)-enantiomer. The latter’s effect on the mice, for each parameter, was comparable to results obtained in the control untreated specimens. The promotive effect of the (+)-halofuginone enantiomer on average myofiber diameter and distribution, with a clear shift to the higher diameter bins, suggests this enantiomer’s capacity to improve myofiber hypertrophy. The reduction of fibrosis by this enantiomer was reflected by an increase in utrophin levels [8] and improved diaphragm histopathology. Together, these findings support the observed increase in motor coordination of the mice. The observed differential effect of the two enantiomers, with higher efficacy for the (+)-halofuginone enantiomer, found in this study, is in line with the results of a study by Linder et al. [36] in which the efficacy of the two enantiomers of halofuginone was tested on the activity of *Cryptosporidium parvum* in the intestinal system. Taken together, the results suggest that when tested individually, the (+)-halofuginone enantiomer has promotive effects on histopathology motor coordination/running time, eventually affecting muscle function in *mdx* mice, whereas its mirror enantiomer has no effect on these parameters. Future studies on the effects of these enantiomers should be performed in other models for MDs (e.g., congenital MD) and maybe even in other diseases in which fibrosis is prominent (e.g., liver fibrosis, scleroderma).

Notably, neither the enantiomers, nor the racemic halofuginone had any toxic effects on cultured myogenic cells. In addition, no effect was observed on the body weight (BW) (data not shown), or mortility of the mice which was nil. Moreover, in cultured C2 cells, LDH levels in the medium were comparable in all treatments, including the control cells, clearly indicating no apparent effect of the compounds on cell toxicity at the doses used. This is in contrast to Linder et al. [36] who demonstrated in-vivo toxicity of the (2*R*, 3*S*)-halofuginone enantiomer [corresponding to the (+)-enantiomer]. The discrepancy between the current work and Linder’s study can be explained by the nature of the compounds used: the dihydrobromide salt used in this study (Figure 1) [37] vs. lactate salt [28]. Moreover, there were differences in the administration methods [intraperitoneal (ip) vs. oral (*per os*)] and doses (from 2–10 mg/kg BW [36] and ~0.5 mg/kg BW here). It should be noted, in terms of the current study, that the comparatively lower doses used are comparable to those which are effective in reducing fibrosis and improving histopathology in *mdx* mice [9]. It may well be that the toxicity of the (+)-enantiomer [i.e., the (2*R*, 3*S*)-enantiomer] is dependent on cell type, where muscle cells are less sensitive than intestinal cells. In addition, the difference between the preparation methods used to obtain the enantiomers may have had an effect on cytotoxicity [36,37]. Further studies on other indications using the separate halofuginone enantiomers are warranted to clarify this issue.

As already noted, the racemic form of halofuginone (i.e., a mixture of equal amounts of the single enantiomers) improves motor coordination and running time of *mdx* mice and histopathology of *mdx* diaphragms [8,9,22]. However, here, for the first time, we show that the effect of the (+)-halofuginone enantiomer exceeds that of the racemic form. Motor coordination in *mdx* mice with this enantiomer is comparatively improved. Similarly, at equivalent doses, the inhibition of fibrosis and the ability to increase utrophin expression levels in the *mdx* mice used in the study are more pronounced for the (+)-enantiomer. In the case of average myofiber diameter, the effect of the (+)-halofuginone enantiomer was slightly higher than that of the racemic halofuginone. This may imply a differential effect of this enantiomer on parameters that are related to fibrosis versus a direct effect on muscle hypertrophy.

The mechanism that lies behind the differential activity of the enantiomers and the relation to halofuginone’s mechanism of action remains to be seen. Recently, we have shown the necessity of the hydroxy group on halofuginone’s piperidinyl ring for halofuginone’s inhibitory effect on fibrosis [9]. The piperidinyl ring was reported to block the activity of inhibit prolyl-tRNA synthase and thereby, which plays a crucial role in amino-acid synthesis [27,28] and probably in collagen synthesis. In addition, halofuginone was shown to inhibit the TGFβ/Smad3 pathway, at least in part, by elevating the activity Akt activity its association with Samd3 [19]. In addition to fibrosis, muscle waste and dysfunction in DMD patients could be due, to severe defects in mitochondrial activity, such as oxidative dysfunction and ATP synthesis, dysregulation of Ca^2+^ homeostasis and increase in reactive oxygen species [42,43,44,45,46]. Recent studies have shown that halofuginone inhibits satellite cell apoptosis in *mdx* mice by affecting members of the Bax family [40]. It may well be that other mitochondrial functions could be ameliorated by halofuginone.

The non-toxicity of the (+)-halofuginone enantiomer at doses that effectively improve muscle histopathology and function, combined with its higher efficacy, support its consideration as a future therapeutic drug for DMD patients. The findings of this study imply that lower doses of the halofuginone (+)-enantiomer can be administered to DMD patients and still be as effective as higher levels of the racemic form. This is of the utmost importance in preventing adverse side effects of higher doses of racemic halofuginone in clinical trials with DMD patients. 

## 4. Materials and Methods

### 4.1. Reagents

Dulbecco’s Modified Eagle’s Medium (DMEM), sera and antibiotic–antimycotic solution were purchased from Biological Industries (Beit-Haemek, Israel). Sirius red F3B was obtained from BDH Laboratory Supplies (Poole, UK). The LDH kit CyQUANT was purchased from Invitrogen (Eugene, OR, USA).

### 4.2. Animals and Experimental Design

Male *mdx* mice [C57BL/10ScSn-Dmdmdx/J (Stock 001801), dystrophin-deficient] (Jackson Laboratories, Bar Harbor, ME, USA) were divided into four groups and injected ip with either saline (Control, *n* = 4), or 10 μg of the optically active halofuginone enantiomers (+) and (−) [(+)-Halo (*n* = 6) and (−)-Halo (*n* = 5), respectively], either alone or as a combination of equal amounts of the enantiomers (racemic molecule, Racemic, *n* = 6), three times per week for 10 weeks, starting at 4 weeks of age. This regime was based on earlier studies [9,22]. The halofuginone enantiomers, (+) and (−)-Halo (Figure 1B,C), were synthesized as described in Smullen and Evans [37] in enantiomeric excesses of 88% and 87% respectively. They were used as their stable dihydrobromide salts. The mice were kept in cages under constant photoperiod (12 L:12 D) with free access to food and water. At the end of the experiment, the mice were subjected to a motor coordination assay. Diaphragm muscles were collected for further analyses. The experiments were carried out according to the guidelines of the Volcani Center Institutional Committee for Care and Use of Laboratory Animals (IL-822/19, date: 19 March 2019).

### 4.3. Motor Coordination Assay

Motor coordination and balance were evaluated with an accelerating single-station RotaRod treadmill (Med Associates Inc., St. Albans, VT, USA) as previously described [22]. The mice were placed one at a time on the rod, which was rotating at an initial speed of 3.5 rpm. The speed was gradually increased from 3.5 to 35 rpm over a period of 5 min, and the time that the mice stayed on the rod was recorded. The mice (*n* = 4–6 mice per group) were subjected to three successive trials in each session, and the test was repeated on 2 consecutive days. The performance of each mouse was measured as the mean of its best individual performances over the three trials on the second day. 

### 4.4. Histology and Fibrosis Evaluation 

Muscle samples were fixed with 4% paraformaldehyde in PBS at 4 °C overnight. They were then dehydrated and embedded in paraffin as previously described [22]. For fibrosis evaluation, the sections (5 μm) were stained with Sirius red as described in Turgeman et al. [22] and analyzed with ImagePro software (Media Cybernetics Inc., Silver Spring, MD, USA). Photographs (*n* = 20, i.e., 20 pictures from randomly selected sections from at least three different mice from each group of mice) were taken for analysis at 20× magnification under a light microscope (Olympus, Hamburg, Germany) with a DP-11 digital camera (Olympus). The results were calculated as red area divided by the total area (red + green) and presented as the proportion of fibrotic muscle area (mean ± SE). Blank areas, assumed to be artefactual, were excluded. There were no statistically significant differences between the mice in the same group. Therefore, all data of the same group were pooled.

### 4.5. Immunohistochemistry 

Paraffin-embedded muscle sections were deparaffinized and rehydrated with ethanol and citrate buffer. The sections were then incubated with 3% normal donkey serum and 10% bovine serum albumin in 0.05% Tween 20–PBS (*v*/*v*) for 1 h at room temperature followed by overnight incubation at 4 °C with polyclonal rabbit anti-utrophin (1:50, Santa Cruz Biotechnology, Dallas, TX, USA) [8]. The secondary antibody was Cy3 goat anti-rabbit IgG (1:200, Jackson Laboratories). Nuclei were then stained with 4′,6-diamidino-2-phenylindole (DAPI, 1:1000). For utrophin detection, microscope observations and image acquisition were performed with a Leica sp8 inverted laser-scanning confocal microscope (Leica Camera, Wetzlar, Germany), equipped with a 405-nm diode laser, 488-nm OPSL, 552-nm OPSL and the HC PL APO CS2 63X/1.40 oil objective. “RED” was excited at 552 nm.

### 4.6. Myofiber Diameter Analysis

Muscle sections were stained with H&E and the myofiber diameter was determined by analyzing the lesser diameters of the myofibers, as described by Dubowitz [47]. At least 10 arbitrary fields in two to three serial sections of each muscle sample from each mouse were photographed under a light microscope with a DP-11 digital camera (Olympus). Myofiber diameter was then determined with Adobe Photoshop software. In each muscle sample, the lesser myofiber diameter was measured for individual myofibers, analyzing between 4300 and 6000 myofibers from four mice per treatment. Counted myofibers per section were averaged per animal and statistically analysed (*n* = 4).

### 4.7. Toxicity Analysis

The cytotoxicity test was conducted using the commercial cytotoxicity assay kit CyQUANT LDH (Invitrogen). The test is based on measuring the activity of LDH leaking from dead/damaged cells to the medium. Thus, presence of LDH in the medium is a good parameter for cytotoxic activity in the treated cells. Briefly, mouse myogenic cell line C2 [39] was grown in 20% fetal bovine serum-containing DMEM (growing medium). C2 cells were plated in triplicate on 24-well plates (9000 cells/well) in growing medium and after 10 h, they were treated with the enantiomers or racemic form of halofuginone at various concentrations for 24 h. Then, 50 μL of the medium in which the cells from each well were treated were transferred to a 96-well plates (*n* = 5). To each well, 50 μL CyQUANT kit Reaction Mix was added and incubated for 30 min in the dark. Then, the reaction was stopped with the kit’s Stop Reaction solution. Absorption was detected at 490 nm with a Synergy-2 Multimode Microplate Reader (BioTek Instruments, Winooski, VT, USA). Percent of cytotoxicity was calculated by the following equation:

(Compound-treated LDH activity − spontaneous LDH activity) × 100

Maximum LDH activity − spontaneous LDH activity

where maximum LDH activity = LDH activity of cells treated with lysis buffer; spontaneous LDH activity = LDH produced with no treatment

### 4.8. Statistical Analysis

The data were subjected to one-way analysis of variance (ANOVA) and to all-pairs Tukey–Kramer HSD test using JMP Pro 14 software (SAS Institute, Cary, NC, USA). 

## 5. Conclusions

The two enantiomers of halofuginone had differential effects on coordination in *mdx* mice, as well as on fibrosis and diaphragm histopathology; the (+)-enantiomer was the most active of the two. Moreover, the (+)-enantiomer’s effects surpassed those of the racemic halofuginone in improving fibrosis and motor coordination, thereby indirectly improving muscle function. At the doses used in the study, no toxic effects were detected. On the basis of these findings it can be concluded that the (+)-enantiomer of halofuginone could serve as a useful future therapeutic drug for DMD patients towards improving their quality of life.

## Data Availability

Not applicable.

## Abbreviations

BW	Body weight
DAPI	4′,6-diamidino-2-phenylindole
DMD	Duchenne muscular dystrophy
H&E	Hematoxylin and eosin
ip	Intraperitoneally
LDH	Lactate dehydrogenase
